# Microstructure and Mechanical Properties of TiB_2_/TiC Particle Modified Al-Mg-Si Alloys Fabricated by Wire-Arc Additive Manufacturing

**DOI:** 10.3390/ma18091978

**Published:** 2025-04-27

**Authors:** Tao Li, Jiqiang Chen, Lingpeng Zeng, Zhanglong Tuo, Jieke Ren, Zuming Zheng, Hanlin Wu

**Affiliations:** 1 School of Material Science and Engineering, Jiangxi University of Science and Technology, Ganzhou 341000, China; 2 Ningbo Boway Alloy Material Co., Ltd., Ningbo 315135, China; 3 State Key Laboratory of Comprehensive Utilization of Low-Grade Refractory Gold Ores, Zijin Mining Group Co., Ltd., Xiamen 361000, China

**Keywords:** Al-Mg-Si alloy, WAAM, particles, mechanical property, microstructure

## Abstract

TiB_2_ and TiC particles were separately introduced to modify the Al-Mg-Si alloy fabricated by wire-arc additive manufacturing (WAAM) to solve the problem of hot cracking. The results showed that modification of the Al-Mg-Si alloy with TiB_2_ or TiC particles completely suppressed the hot cracks found in commercial Al-Mg-Si alloys fabricated by WAAM due to the transformation from columnar grains to fine equiaxed grains with a mean diameter of approximately 10 μm. The ultimate strength and yield strength of the as-deposited Al-Mg-Si/TiB_2_ (AD-TB) and Al-Mg-Si/TiC (AD-TC) alloys were similar, but the elongation of the latter one was higher due to its low porosity. The ultimate strength (353.7 ± 5.0 MPa) and yield strength (309.7 ± 1.9 MPa) of the heat-treated Al-Mg-Si/TiC (HT-TC) alloy was significantly higher than those (300.8 ± 2.7 MPa and 256.2 ± 2.8 MPa, respectively) of the heat-treated Al-Mg-Si/TiB_2_ (HT-TB) alloy. The fatigue resistance of the HT-TC was better than that of the HT-TB due to less porosity and a more uniform distribution of TiC particles in the HT-TC alloy.

## 1. Introduction

As a rapid forming technology for metal parts, wire-arc additive manufacturing (WAAM) technology has the advantages of cost-effectiveness, rapid manufacturing rates and enhanced material efficiency [1,2]. It has attracted significant attention in the manufacturing industry due to these advantages and has emerged as a transformative approach for producing large-scale aluminum components in the aerospace and transportation sectors–particularly for lightweight parts [3,4,5]. Recent advancements in WAAM of high-strength Al alloys have mainly focused on Al-Cu [6], Al-Mg [7,8], and Al-Zn-Mg-Cu [9] alloys. Al-Mg-Si alloys have advantages such as moderate strength, good corrosion resistance and a good oxidation effect [10]. Therefore, it is necessary to promote the application of WAAM technology for Al-Mg-Si alloys.

Hot cracking constitutes a major impediment to the development of WAAM for Al-Mg-Si alloys [11,12]. Extensive research has been conducted on the issue of hot cracks [13,14,15]. The reason for the occurrence of hot cracks is that the liquid metal with high purity crystallizes first, and the metal that crystallizes later contains more impurities, which are enriched at the grain boundaries. Meanwhile, an eutectic with a low melting point is formed, creating a liquid film between the grains. If tensile stress is applied, cracks will occur [16,17]. In general, the formation of hot cracks is a result of the combined action of the liquid film and the tensile stress. Solving the hot crack problem of Al-Mg-Si alloys is necessary to promote the application of WAAM technology for Al-Mg-Si alloys.

Based on the research progress of WAAM manufacturing high-strength Al alloys in recent years, there are several ways to suppress hot cracks: (1) Adding alloy elements or effective nucleation particles, which can reduce the tendency of dendrite growth during solidification and form a fine equiaxed grain structure [18,19,20,21]. The main reason is that, on the one hand, the transformation from columnar grains to equiaxed grains shortens the feeding channel of liquid metal, making it easier to backfill the liquid metal; On the other hand, an increase in grain boundary density is beneficial for the transmission and dispersion of residual stresses, thereby significantly reducing the local stress concentration and suppressing crack formation. For example, Yuan et al. [20] prepared a TiN-particle modified Al-Zn-Mg-Cu alloy by the WAAM process, eliminating the coarse columnar grains, randomizing the orientations, and reducing the average grain size from 459 nm to 104 nm. Klein et al. [21] demonstrated TiB_2_-modified Al-Mg-Si alloy WAAM deposits free from macroscopic cracks, exhibiting fine equiaxed grains (<30 μm) without a crystallographic texture. (2) Optimizing the process parameters in WAAM to control heat input, which can reduce temperature gradients and thermal stresses to suppress cracks. For example, Doumenc et al. [22] fabricated crack-free 6061 alloy components by increasing the WAAM welding speed, while Ma et al. [23] proposed programmable thermal input WAAM for efficient low-heat-input crack-free fabrication of Al-Mg alloys. (3) Introducing interlayer friction stir processing (IFSP) during the WAAM process, which can enhance the microstructural homogeneity and eliminate cracks [24]. For example, Guo et al. [24] compared the microstructure and mechanical properties of WAAM-T6 thin-walled components with those of WAAM + IFSP-T6 thin-walled components. The samples subjected to IFSP exhibited a crack-free microstructure, and their tensile strength increased by 223%.

In contrast to conventional reinforcement strategies utilizing particles such as SiC or Al_2_O_3_, TiB₂ and TiC demonstrate superior performance in grain refinement and strength enhancement, attributed to their distinctive physicochemical characteristics. This advancement successfully resolves the long-standing strength–ductility trade-off associated with crack mitigation in conventional approaches, providing a materials engineering solution balancing scientific rigor and industrial viability for WAAM-fabricated lightweight components. In this work, TiB_2_ and TiC particles were separately introduced to modify a Al-Mg-Si alloy fabricated by WAAM to solve the problem of hot cracking. The microstructures, mechanical properties and fatigue resistances of the as-deposited and heat-treated Al-Mg-Si/TiB_2_ and Al-Mg-Si/TiC alloys were investigated and compared.

## 2. Experimental Methods

The welding wires of an Al-Mg-Si alloy, Al-Mg-Si alloy with the addition of submicron sized TiB_2_ particles and Al-Mg-Si alloy with the addition of nano-sized TiC particles were used as filler metal for wire-arc additive manufacturing. TiB_2_ particles were introduced in the form of an Al-2.3Ti-1B master alloy, while TiC nanoparticles were introduced in the form of an Al-5wt%TiC master alloy during the melting process. The nominal chemical compositions of the welding wires are shown in Table 1, and the corresponding printing parameters of the WAAM process are shown in Table 2. One piece of thin-walled sample was fabricated for each material.

The microstructures of the Al-Mg-Si alloy, Al-Mg-Si/TiB_2_ alloy (denoted as TB) and Al-Mg-Si/TiC alloy (denoted as TC) fabricated by WAAM were analyzed by optical microscope (OM), field emission scanning electron microscopy (FE-SEM) and transmission electron microscopy (TEM). The samples for OM were ground with sandpaper from 600 to 2000 grits, and then polished with polishing paste. Subsequently, these samples were observed via an optical microscope (ZEISS Axioskop.A1, Zeiss, Oberkochen, Germany). The samples for SEM were prepared by mechanical grinding and electrolytic polishing processes, and then were subjected to morphology observation, energy dispersive spectroscopy (EDS) and electron backscatter diffraction (EBSD) analysis on a field emission scanning electron microscope (FE-SEM, ZEISS ∑IGMA, Zeiss, Oberkochen, Germany). The samples for TEM were ground with sandpaper to a thickness less than 100 μm, and thinned in a double-jet thinning solution with a HNO_3_:CH_3_OH ratio of 3:7 using a double-jet thinning instrument (model MTP-1A, Jiaoda, Shanghai, China). The double-jet temperature was controlled at −25 °C to −40 °C, and the double-jet voltage was 20 V. After thinning, the samples were cleaned in alcohol and then air-dried naturally. The prepared samples were observed using an FEI Tecnai G2 F20 TEM (FEI, Hillsboro, OR, USA).

The mechanical properties of the Al-Mg-Si alloy, TB and TC components fabricated by WAAM were evaluated by hardness tests and tensile tests. The samples for the hardness tests were ground with sandpaper and polished to ensure the surface was smooth and flat. The hardness test was carried out on a hardness tester (200HVS-5, Huayin, Yantai, China) according to GB/T 4340.1 [25], where the load was 1 kg and the holding time was 15 s. The average value and error were calculated based on more than five measured results. Solution treatment was applied prior to aging to dissolve the primary second phase into the aluminum matrix and achieve a supersaturated solid solution. The aging hardening curves of the Al-Mg-Si alloy were determined at an aging temperature of 175 °C after solution treatment at 530 °C for 1 h, and hardness at the aging time of 12 h reached the peak value, i.e., the T6-treatment (also denoted as T6). The as-printed samples and peak-aged samples along the travelling direction (0° direction) were prepared for tensile tests. The size of the tensile samples was calculated according to GB/T 228.1 [26], where the thickness of the tensile sample was 3 mm and the gauge length was 30 mm. The tensile samples were polished with 600-grit sandpaper to ensure the surface was smooth and flat. The tensile tests were carried out with a CT5105 testing machine (SUNS, Shenzhen, China), and the tensile speed was set at 1 mm/min. A 25 mm extensometer was used to measure the strain generated during the tensile process. The average values of the tensile results of four samples from each alloy were taken as the final tensile result, and the error was calculated. The three alloys were all sampled at similar locations of the components.

## 3. Results

### 3.1. Microstructure of the As-Deposited Alloys

**Figure 1:**
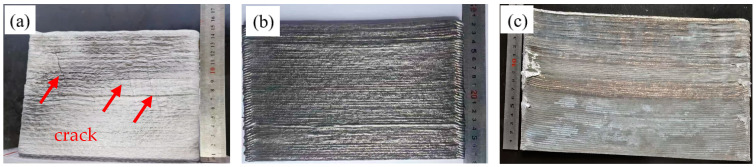
Thin-walled alloy components prepared by WAAM: (**a**) Al-Mg-Si, (**b**) TB, (**c**) TC.

Figure 1 shows the macroscopic morphologies of the Al-Mg-Si alloy, TB and TC prepared by WAAM. It shows distinct cracks in the Al-Mg-Si alloy (indicated by red arrows), while no cracks are observed in the TB or TC.

**Figure 2:**
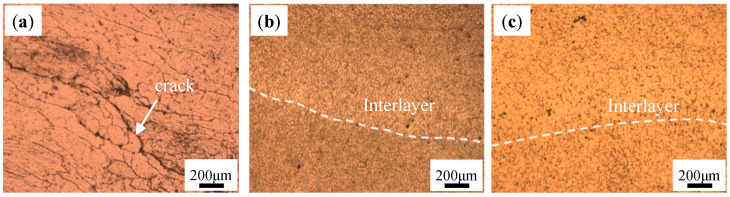
OM microstructure: (**a**) Al-Mg-Si, (**b**) TB, (**c**) TC.

**Figure 3:**
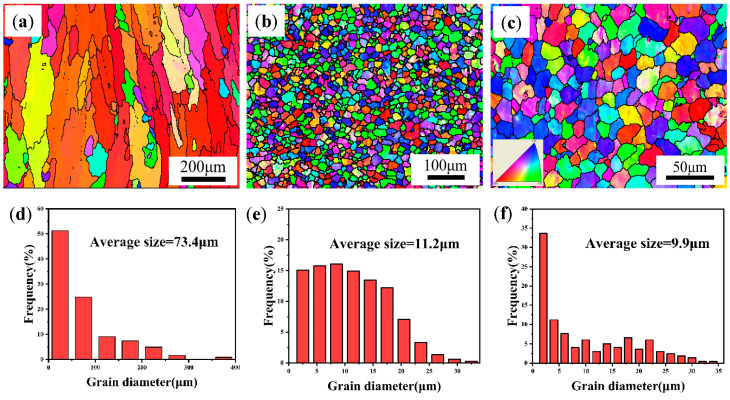
EBSD images and corresponding grain size distributions of three alloys: (**a**,**d**) Al-Mg-Si alloy, showing the columnar grains with an average grain size of 73.4 μm; (**b**,**e**) TB, showing the fine equiaxed grains with an average grain size of 11.2 μm; (**c**,**f**) TC, showing the fine equiaxed grains with an average grain size of 9.9 μm.

Figure 2 shows the OM microstructure of the as-deposited Al-Mg-Si alloy (denoted as AD Al-Mg-Si alloy), as-deposited Al-Mg-Si/TiB_2_ alloy (denoted as AD-TB) and as-deposited Al-Mg-Si/TiC alloy (denoted as AD-TC) manufactured by WAAM. It can be found that the AD Al-Mg-Si alloy has several micro-cracks throughout the whole image, which is consistent with the results of Figure 1a. For AD-TB and AD-TC, the grain microstructure is composed of fine equiaxed grains due to the addition of particles that act as the nucleus of heterogeneous nucleation. Figure 3 shows the EBSD images and corresponding grain size distributions of the AD Al-Mg-Si alloy, AD-TB and AD-TC. It clearly illustrates the comparison between the columnar grains in the AD Al-Mg-Si alloy (Figure 3a) and fine equiaxed grains in the AD-TB (Figure 3b) and AD-TC (Figure 3c). The average grain size of the AD Al-Mg-Si alloy is about 73.4 μm, while those of the AD-TB and AD-TC are very close, around 11.2 μm and 9.9 μm, respectively. The formation of fine equiaxed grains can reduce the thermal stress caused by solidification shrinkage and reduce the possibility of hot cracking, and therefore suppress the occurrence of the hot cracks that are found in the original Al-Mg-Si alloy.

**Figure 4:**
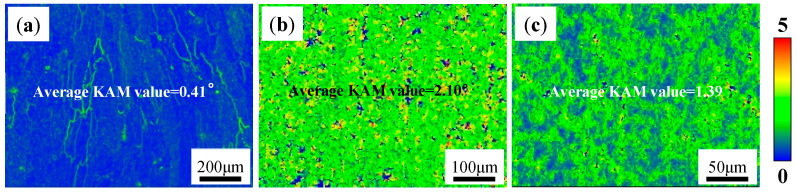
KAM distributions of three as-deposited alloys: (**a**) Al-Mg-Si alloy, showing the average KAM value of 0.41°; (**b**) TB, showing the average KAM value of 2.10°; (**c**) TC, showing the average KAM value of 1.39°.

Figure 4 shows the Kernel Average Misorientation (KAM) value distribution of the AD Al-Mg-Si alloy, AD-TB and AD-TC. The KAM values for the AD Al-Mg-Si alloy, AD-TB and AD-TC are measured as 0.41°, 2.10°, and 1.39°, respectively. The local orientation difference at the grain boundary of the AD Al-Mg-Si alloy is high, while the AD-TB and AD-TC alloys exhibit more uniform local misorientation distributions. Moreover, the KAM value can be approximated as the dislocation angle (*ϑ*) to calculate the geometrically necessary dislocation density ($\rho_{GND}$). The calculation formula is as follows [27]:

(1) $$\rho_{GND}=\frac{2\vartheta }{ub}$$

where *u* is the unit length and its value is 1.5 μm and *b* is the Burgers vector and its value is 0.286 nm. The $\rho_{GND}$ of the AD Al-Mg-Si alloy, AD-TB and AD-TC are 3.24 × 10^13^/m^2^, 1.66 × 10^14^/m^2^ and 1.09 × 10^14^/m^2^, respectively. A higher $\rho_{GND}$ in alloys indicates greater residual stress [28]. These results show that the residual stress of the AD-TB and AD-TC alloys is similar and larger than that of the AD Al-Mg-Si alloy. The reason may be associated with the Al-Mg-Si alloy generating hot cracks during the solidification process and releasing most of the residual stress.

**Figure 5:**
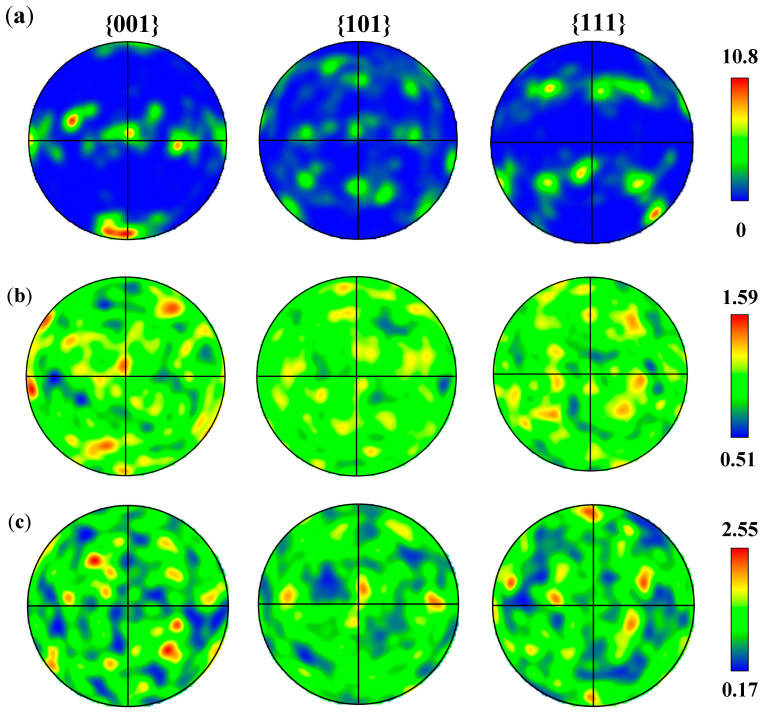
Polar figures of three as-deposited alloys: (**a**) Al-Mg-Si alloy, (**b**) TB, (**c**) TC.

Figure 5 shows the polar figure of the AD Al-Mg-Si alloy, AD-TB and AD-TC. The results show that the maximum texture strength of the AD Al-Mg-Si alloy, AD-TB and AD-TC are 10.8, 1.59 and 2.55, respectively. Figure 5a reveals distinct <111> and <100> fiber textures in the Al-Mg-Si alloy, indicating the formation of preferential crystallographic orientations along the deposition direction during rapid solidification of the additive manufacturing process. On the other hand, AD-TB and AD-TC did not show significant preferred orientations (Figure 5b,c).

**Figure 6:**
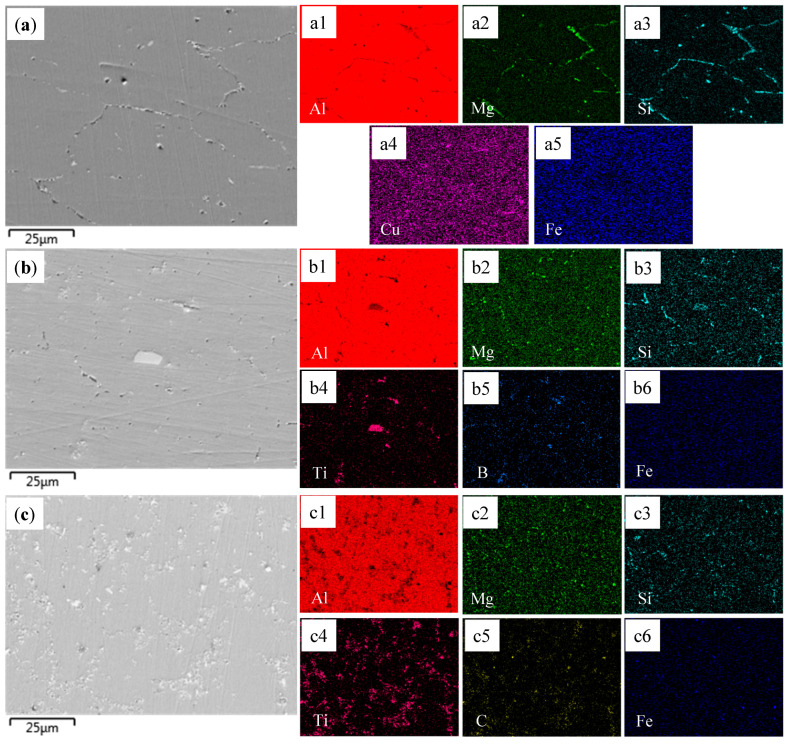
SEM images and corresponding EDS of as-deposited alloys: (**a**) Al-Mg-Si alloy; (**a1**–**a5**) Al, Mg, Si, Cu, Fe; (**b**) TB; (**b1**–**b6**) Al, Mg, Si, Ti, B, Fe; (**c**) TC; (**c1**–**c6**) Al, Mg, Si, Ti, C, Fe.

Figure 6 shows the SEM microstructure morphology of the as-deposited Al-Mg-Si, TB and TC alloys. For the Al-Mg-Si alloy (Figure 6a), pronounced segregation of Mg and Si is observed at the grain boundaries, forming a coarse primary Mg_2_Si phase, while Cu remains uniformly dispersed in the α-Al matrix. In contrast, the introduction of TiB_2_ or TiC particles significantly homogenizes the precipitate distribution. As shown in Figure 6b,c, the reinforcing particles act as preferential nucleation sites for fine Mg-Si precipitates, suppressing solute segregation along the grain boundaries.

**Figure 7:**
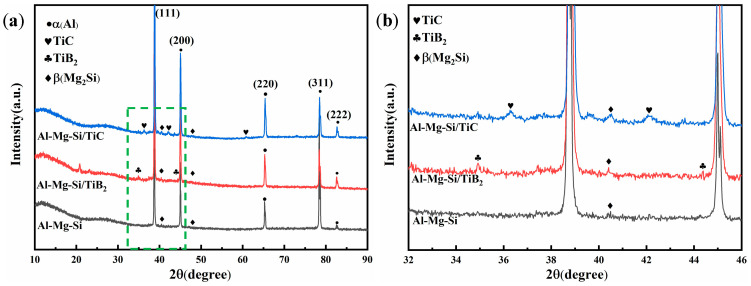
(**a**) The XRD results of three as-deposited alloys; (**b**) the zoom area corresponding to the green box of (**a**).

Figure 7 shows the XRD patterns of three as-deposited alloys. The dominant α(Al) peaks and the primary β(Mg_2_Si) phase peaks were found in the as-deposited Al-Mg-Si alloy. Meanwhile, in addition to the α(Al) peaks and the primary β(Mg_2_Si) phase peaks, the TiB_2_ phase peak (PDF#75-0967) and TiC phase peak (PDF#32-1383) were also evident in the TB and TC (Figure 7b), respectively. The peak intensity of these two modified particle phases was relatively low due to their low content.

**Figure 8:**
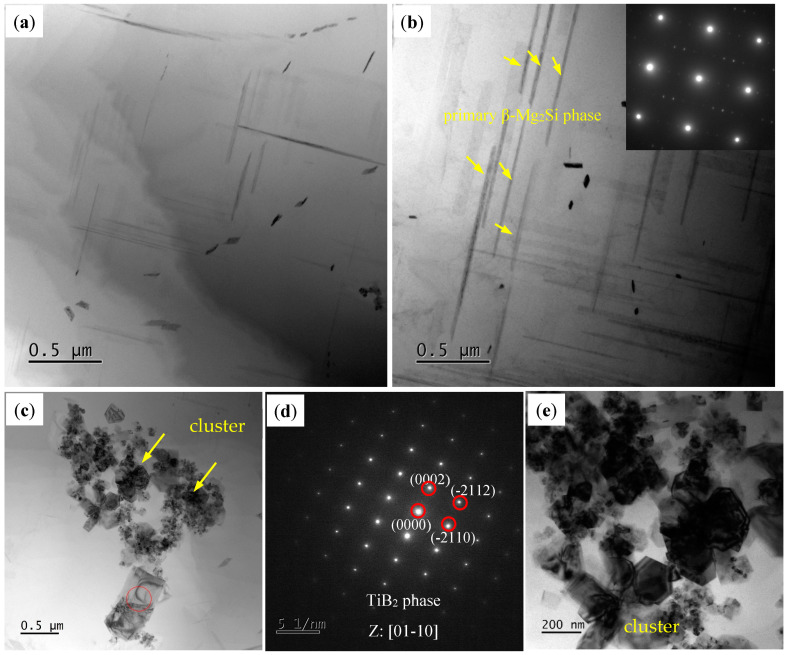
TEM images of AD-TB alloy: (**a**,**b**) morphology at grain boundaries; (**c**) morphology of the internal grains; (**d**) SEAD of (**c**); (**e**) TiB_2_ particles are reunited.

Figure 8 shows TEM images of the AD-TB. The needle-shaped second phase in Figure 8a,b could be identified as the primary β-Mg_2_Si phase, evaluated by the corresponding selected area electron diffraction (SEAD) pattern and by the result of the XRD pattern. The size of these primary β-Mg_2_Si phases is very large, and the length of most phases are above 1 μm. In addition, there are also some other particles in the grain of the AD-TB as shown in Figure 8c. Large numbers of these particles gathered together to form a cluster, and the size of these particles ranged from 50 nm to 1000 nm, as speculated by Figure 8e, which is the zoom area of Figure 8c. The SEAD pattern of these particle is shown in Figure 8d, and the lattice constant as well as the crystal structure of these particles is in good agreement with the TiB_2_ phase (PDF#75-0967), which is also found in the XRD result (Figure 7). This suggests that the added TiB_2_ particles are not dispersed in the alloy, which may have adverse effects on the properties of the alloy.

**Figure 9:**
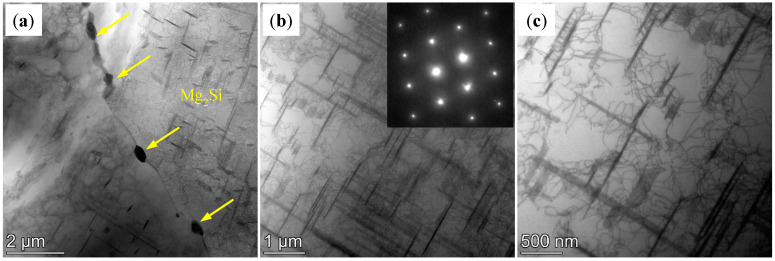
TEM images of the AD-TC: (**a**) BF image showing the precipitates on the grain boundary and in the grain; (**b**,**c**) BF image and corresponding SAED pattern showing the precipitates in the grain.

**Figure 10:**
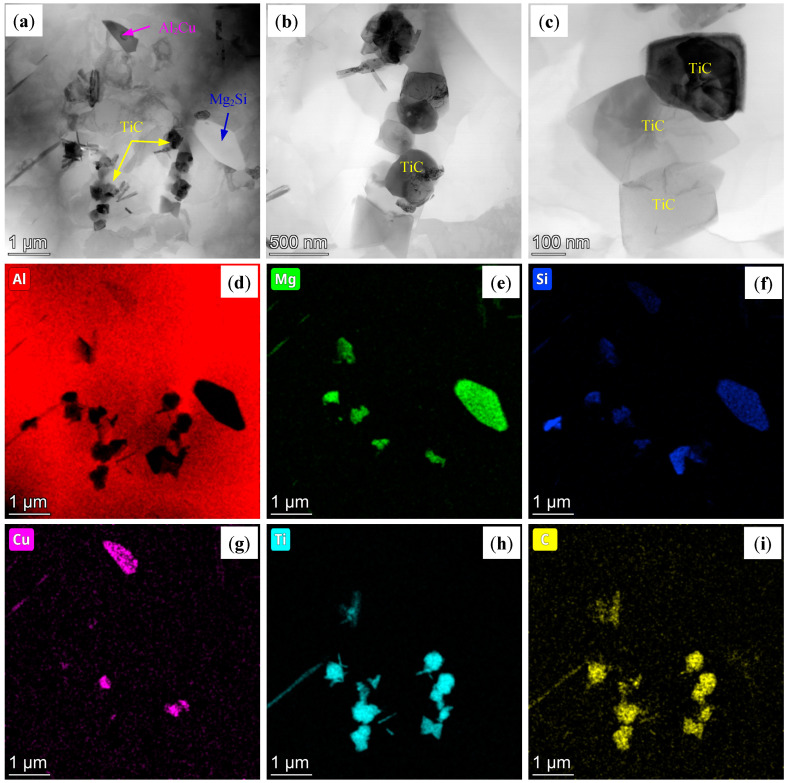
(**a**) TEM images of the AD-TC alloy showing different second phases; (**b**,**c**) the morphology and size of the TiC particles; (**d**–**i**) EDS mapping corresponding to (**a**) for Al, Mg, Si, Cu, Ti and C, respectively.

On the other hand, Figure 9 shows TEM images of AD-TC. Similar to AD-TB, there are also some primary β-Mg_2_Si phases with a large size on the grain boundary and in the grain of the AD-TC. In addition, dislocations were evident in the AD-TC, and the number density of its dislocations was higher than that of the dislocations in the AD-TB. Figure 10 shows the TEM images and the corresponding EDS mapping of the AD-TC to show different second phases. The results of the EDS mapping (Figure 10d–i) suggest that there are Al_2_Cu phase and TiC particles in addition to the primary β-Mg_2_Si phase. Figure 10b,c illustrate the morphology of the TiC particles. Compared to the TiB_2_ particles in the AD-TB, the TiC particles in the AD-TC are more dispersed, and the size of the TiC particles is about 100 nm to 200 nm.

### 3.2. Microstructure of the T6-Treated Alloys

**Figure 11:**
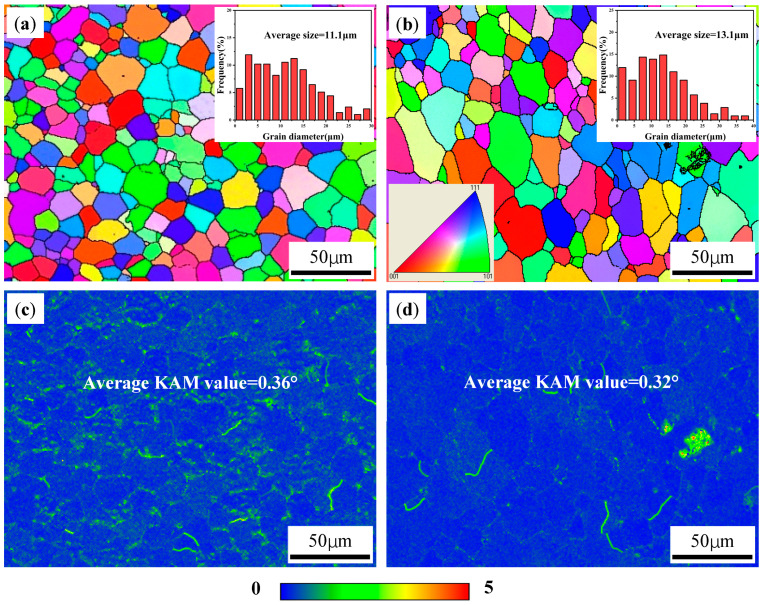
EBSD images, corresponding grain size distributions and KAM distributions of two T6-treated alloys: (**a**,**c**) TB, showing the fine equiaxed grains with an average grain size of 11.1 μm and average KAM value of 0.36°; (**b**,**d**) TC, showing the fine equiaxed grains with an average grain size of 13.1 μm and average KAM value of 0.32°.

Figure 11 shows the EBSD images, corresponding grain size distributions and KAM distributions of T6-treated Al-Mg-Si/TiB_2_ alloy (denoted as T6-TB) and T6-treated Al-Mg-Si/TiC alloy (denoted as T6-TC). The result indicates that the average grain sizes of the T6-TB and T6-TC alloy were 11.1 μm and 13.1 μm, respectively, which are very close to the corresponding as-deposited alloys. This implies that the effect of T6 heat treatment on the grain size of the as-deposited alloys is not significant. In addition, the average KAM values of the T6-TB and T6-TC are 0.36° and 0.32°, respectively, which are significantly lower than those of the corresponding as-deposited alloys. Moreover, the geometric necessary dislocation densities (ρ_GND_) of the T6-TB and T6-TC are 2.85 × 10^13^/m^2^ and 2.53 × 10^13^/m^2^, respectively, which are also significantly lower than those of the corresponding as-deposited alloys. This result also supports the hypothesis that the heat treatment alleviated the local residual stress of the alloys [28].

**Figure 12:**
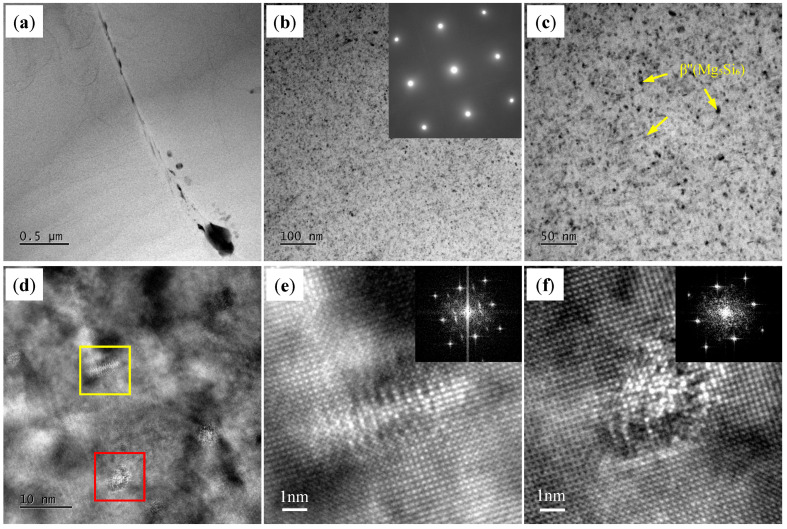
TEM images of the T6-TB: (**a**) BF image showing the precipitates on the grain boundary and in the grain; (**b**,**c**) BF image and corresponding SAED pattern showing the precipitates in the grain; (**d**–**f**) HETEM images of the precipitates and the corresponding FFT. Figure 12e,f are the zoom area of yellow box and red box in Figure 12d, respectively.

**Figure 13:**
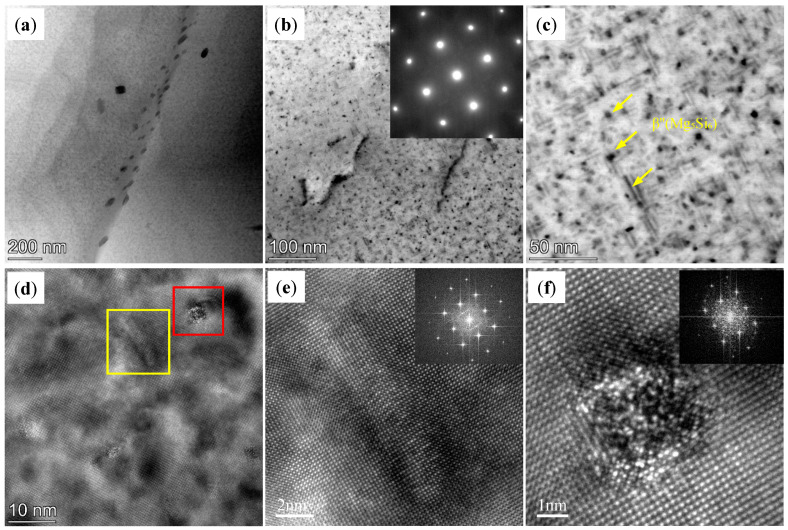
TEM images of the T6-TC: (**a**) BF image showing the precipitates on the grain boundary and in the grain; (**b**,**c**) BF image and corresponding SAED pattern showing the precipitates in the grain; (**d**–**f**) HETEM images of the precipitates and the corresponding FFT. Figure 13e,f are the zoom area of yellow box and red box in Figure 13d, respectively.

Figure 12 shows the TEM images of the T6-TB alloy. There are many dense, fine needle-like and spherical precipitates in T6-TB, which are identified as the metastable β″ (Mg₅Si₆) phase with an average size of 28.4 ± 3 nm. The phase identification is also further supported by the high-resolution TEM (HRTEM) images (Figure 13d–f) and corresponding Fast Fourier Transform (FFT) patterns. The indexed images exhibited remarkable consistency with the precipitation characteristics previously reported by Zhao et al. [29]. Figure 13 shows the TEM images of T6-TC. Similar to T6-TB, there are also many dense, fine needle-like and spherical precipitates with an average size of 18.1 ± 2.5 nm in T6-TC. These precipitates are also identified as the metastable β″ (Mg₅Si₆) phase by the SEAD pattern, HRTEM and corresponding Fast Fourier Transform (FFT).

### 3.3. Mechanical Properties

**Figure 14:**
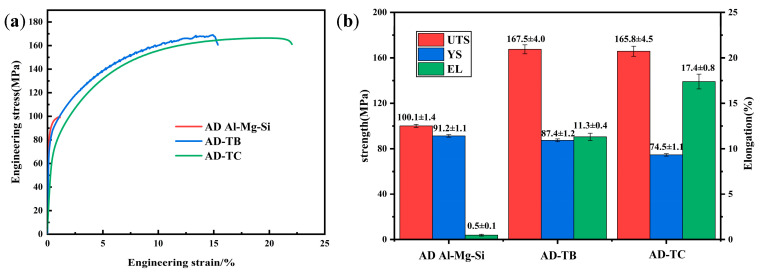
Stress–strain curves and the mechanical property results of three as-deposited alloys: (**a**) stress–strain curves, (**b**) mechanical property results.

Figure 14 shows the stress–strain curves and the mechanical property results of three as-deposited alloys. Due to the presence of severe crack defects, the Al-Mg-Si alloy exhibits anomalous mechanical properties with relatively low ultimate tensile strength (UTS) and elongation (EL). Moreover, the UTS of TB and TC are very close, while TC demonstrates significantly higher EL than TB. The UTS, yield strength (YS) and EL of TB are 167.5 ± 4.0 MPa, 87.4 ± 1.2 MPa and 11.3 ± 0.4, respectively, while those of TC are 165.8 ± 4.5 MPa, 74.5 ± 1.1 MPa and 17.4 ± 0.8, respectively. The detailed mechanical property result values are shown in Figure 14b.

**Figure 15:**
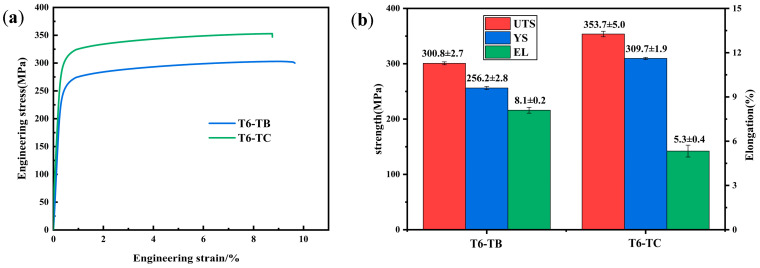
Stress–strain curves and the mechanical property results of two T6-treated alloys: (**a**) stress–strain curves, (**b**) mechanical property results.

Due to the presence of the cracks in the AD Al-Mg-Si alloy, the solution and aging treatment were conducted only on TB and TC. Figure 15 shows the stress–strain curves and the mechanical property results of T6-TB and T6-TC. They suggest that the UTS (353.7 ± 5.0 MPa) and YS (309.7 ± 1.9 MPa) of T6-TC are higher than those (300.8 ± 2.7 MPa and 256.2 ± 2.8 MPa, respectively) of T6-TB, but the EL (5.3 ± 0.4%) of T6-TC is lower than that (8.1 ± 0.2%) of T6-TB. The detailed mechanical property results are shown in Figure 15b. Compared with the as-deposited alloys, the increase in strength and decrease in elongation of the two T6-treated alloys are attributed to the precipitation strengthening caused by the precipitation of precipitates during the T6 treatment. The difference in the mechanical properties between T6-TB and T6-TC should be associated with the added particles, which will be discussed in detail in the next section.

In addition, Table 3 summarizes the comparison of mechanical properties (UTS, YS, and EL) of WAAM-fabricated Al-Mg-Si alloys between this work and previous works. It implies the superiority of the UTS and YS for the TiC particle modified Al-Mg-Si alloy and a good combination of strength and ductility for the TiB_2_ particle modified Al-Mg-Si alloy in this work. It is worth noting that the high strength and elongation of the Al-Mg-Si alloy reported by reference [24] were mainly attributed to the introduction of a friction stir technology between layers.

### 3.4. Fracture Morphology

**Figure 16:**
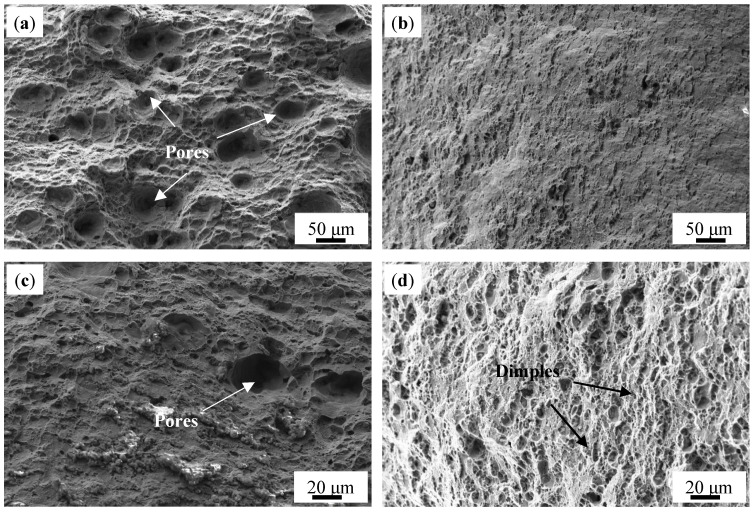
Fracture morphology of the as-deposited alloys: (**a**,**c**) TB, (**b**,**d**) TC.

Figure 16 shows the fracture morphologies of AD-TB and AD-TC. It can be found that there are numbers of pore defects on the fracture surface of AD-TB (Figure 16a), while there are almost no pores on the fracture surface of AD-TC (Figure 16b). High porosity defects in AD-TB should be the main reason why the elongation of AD-TB is lower than that of AD-TC. In addition to the pores, a high density of fracture dimples is also evident in both alloys (Figure 16c,d), which indicates that these two alloys exhibit ductile fractures and good plasticity.

**Figure 17:**
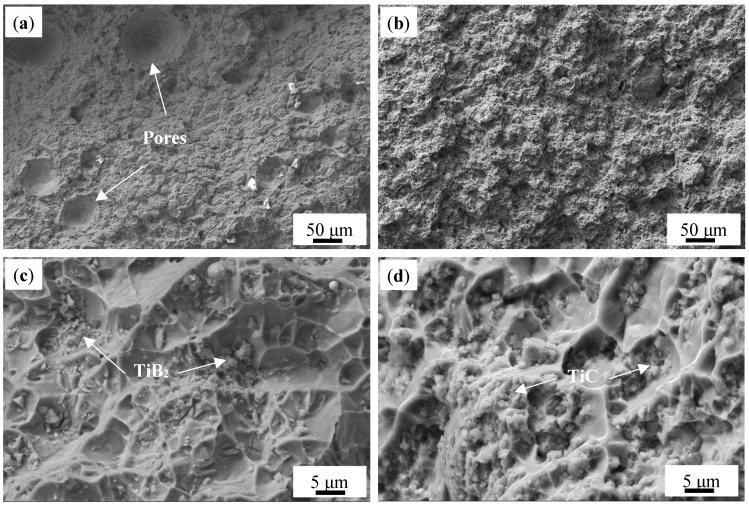
Fracture morphology of the T6-treated alloys: (**a**,**c**) TB, (**b**,**d**) TC.

Figure 17 shows the fracture morphologies of T6-TB and T6-TC. The dimples were shallower for the T6-treated alloys compared to the as-deposited alloys. Moreover, the fracture surface of T6-TC is rougher than that of T6-TB (Figure 17a,b), and is somewhat close to intergranular fractures. Higher magnification fracture morphologies (Figure 17c,d) indicate that there are also some fine particles on the fracture surfaces of the two alloys, which may be associated with the precipitates. The shallower dimples and these fine particles in the T6-treated alloys is also evidence that the elongation of the T6-treated alloy is lower than that of the as-deposited alloys. In addition, the number and density of the dimples on the fracture surface of T6-TB is higher than that of the dimples on the fracture surface of T6-TC. This suggests that the plasticity of T6-TB is better than that of T6-TC, which is in good agreement with the results of the mechanical properties shown in Figure 15.

### 3.5. Fatigue Crack Propagation Behavior

**Figure 18:**
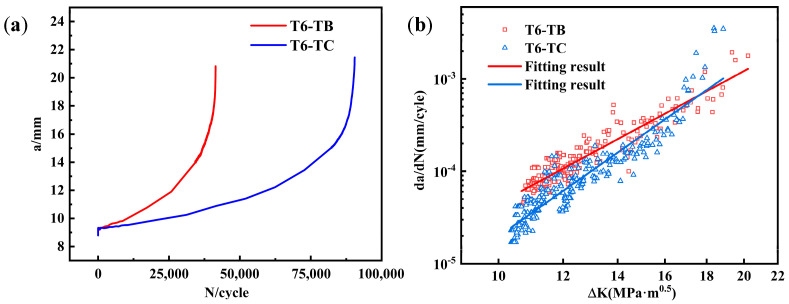
(**a**) The relationship between crack length (**a**) and fatigue cycle times (N) of two T6-treated alloys (*a*-*N* curve); (**b**) Comparison of fatigue crack propagation rates of the two alloys.

The relationship between crack length (*a*) and fatigue cycle times (*N*) of the two T6-treated alloys was plotted as shown in Figure 18a. It can be found that the fatigue life of T6-TB (4.14 × 10^4^ cycles) is close to but lower than that of T6-TC (9.05 × 10^4^ cycles). The critical crack sizes of T6-TB and T6-TC are very close, which are 20.8 mm and 21.5 mm, respectively. These results indicate that the fatigue resistance of T6-TC is better than that of T6-TB.

Fatigue crack propagation is generally divided into three stages according to its propagation rate: low speed propagation zone (I), stable propagation zone (II) and fast propagation zone (III). Figure 18b shows the relationship between the crack growth rate *da*/*dN* and stress intensity factor range Δ*K*, which can be fitted using a Paris model [30]:

(2) $$da/dN={C(\Delta K)}^{m}$$

where *C* and *m* are material constants. It can be found that the fatigue crack growth rate of T6-TB is always higher than that of T6-TC under the same Δ*K*. Under the condition of Δ*K* = 17.7 MPa·m^1/2^, the fatigue crack growth rate of T6-TB reached the minimum value, while that of T6-TC reached the maximum value. Generally, the internal stress has a greater influence on fatigue crack growth in the lower Δ*K* range [31]. According to the KAM value shown in Figure 11 and the calculated geometrically necessary dislocation density, the stress of T6-TB was greater than that of T6-TC. Therefore, when the Δ*K* range is low, the stress of T6-TB is higher than that of T6-TC. The crack growth rate of T6-TB is higher than that of T6-TC. Table 4 shows the parameters fitted by a Paris model and the fatigue crack growth threshold Δ*K_th_* (the value of Δ*K* when *da*/*dN* = 10^−7^ mm/cycle). It suggests that the fitting correlation coefficients *R* are 0.926 and 0.934 respectively, indicating that the fitting curve is reliable. The Δ*K* corresponding to the first experimental data of the two alloys are 10.35 and 10.87, both of which are greater than the values of Δ*K_th_*, indicating that the fatigue crack growth region corresponding to the curve is the stable expansion region (II).

**Figure 19:**
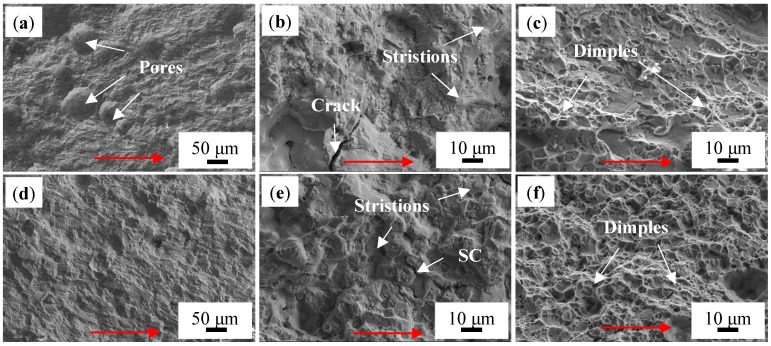
Fatigue fracture morphology of two T6-treated alloys in different stages: (**a**–**c**) TB, (**d**–**f**) TC (the red arrows show the direction of crack growth).

Figure 19 shows the fatigue fracture morphology of the two T6-treated alloys in different stages. Figure 19a,d show the fracture morphology of the low-speed expansion zone (I) of T6-TB and T6-TC, respectively. It can be found that the number of pores in T6-TB is significantly higher than that in T6-TC. This should be one of main factors that increases the rate of fatigue crack propagation of T6-TB. The presence of pores creates localized stress concentration zones within the material, providing preferential sites for crack initiation [32,33]. Following crack initiation, the local stress field around the pores can deflect the crack propagation direction, alter the crack path, and thereby accelerate the propagation rate, ultimately reducing the fatigue life [34]. Figure 19b,e show the fracture morphology in the stable expansion zone (II) of two T6-treated alloys, and they indicate that there are some fatigue striations in both alloys. In addition, there are also some fatigue cracks on the fracture surface of T6-TB with a size greater than 20 μm, which are the main cracks that eventually lead to fracture of the material. The T6-TC fracture has some secondary cracks (SC) with a size of 10–15 μm, and the existence of secondary cracks can absorb part of the energy and slow down the growth of the main cracks. Figure 19c,f show the fracture morphology in the fast propagation zones (III) of the two alloys. Compared with other regions, the fracture morphology in this zone is rougher, mainly dominated by dimples, and there are more fine particles in the dimples, which will lead to easier propagation of fatigue cracks in this region. T6-TB has a small number of shallow fracture dimples, but the T6-TC specimens have fewer fracture defects and more and deeper dimples, which also confirms that T6-TC has better fatigue resistance. Comparative fractographic analysis reveals that pores in the T6-TB alloy accelerate fatigue crack propagation, whereas TiC particles in the T6-TC alloy induce interfacial delamination to form multi-level secondary cracks. This reduces the driving force at the main crack tip, significantly retarding propagation and notably enhancing crack tip shielding effects.

## 4. Discussion

### 4.1. Comparison of the Grain Refinement Mechanism of the Alloys Modified by Two Different Particles

**Figure 20:**
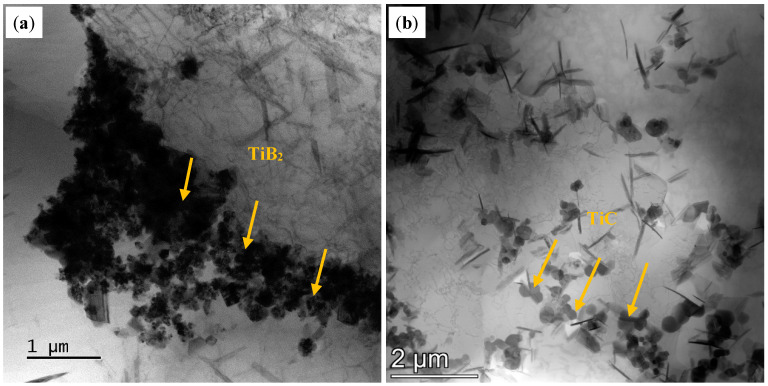
(**a**) The TiB_2p_ distributed at the grain boundary and (**b**) the TiC_p_ inside the grain.

The results of Figure 3 indicate that the alloy grains were significantly refined under the effect of the modification with TiB_2_ and TiC particles. The refining mechanism of TiB_2_ particles is well studied by previous studies, which suggested that TiB_2_ particles have a high lattice matching degree with the α-Al matrix, serving as an efficient hetero-nucleation point of α-Al, promoting nucleation and refining grains [35,36]. However, most of the TiB_2_ particles were found to be enriched at the grain boundaries in the present work (as shown in Figure 20a), and the grain refinement mechanism of TiB_2_ particles may also be associated with hindering of grain boundary migration [37]. This enrichment facilitates Zener pinning, significantly inhibiting grain boundary migration [38]. The pinning effect retains stability even under elevated-temperature thermal cycling, thereby effectively impeding grain coarsening and maintaining a refined equiaxed grain structure [39]. For the grain refinement mechanism of TiC particles, it is reported that the lattice matching degree between TiC and α-Al is low, and the heterogeneous nucleation efficiency is low [19,40]. Meanwhile, there are some works that have shown that nano-sized TiC particles can also act as heterogeneous nucleation sites to refine the grain [41]. The nano-sized surface curvature of TiC particles significantly reduces the nucleation energy barrier, enabling them to act as high-density heterogeneous nucleation sites within the melt, thereby promoting the formation of fine intragranular equiaxed crystals [42]. Additionally, uniformly dispersed TiC particles restrict grain growth by hindering dislocation motion and inducing lattice distortion [43]. 

**Figure 21:**
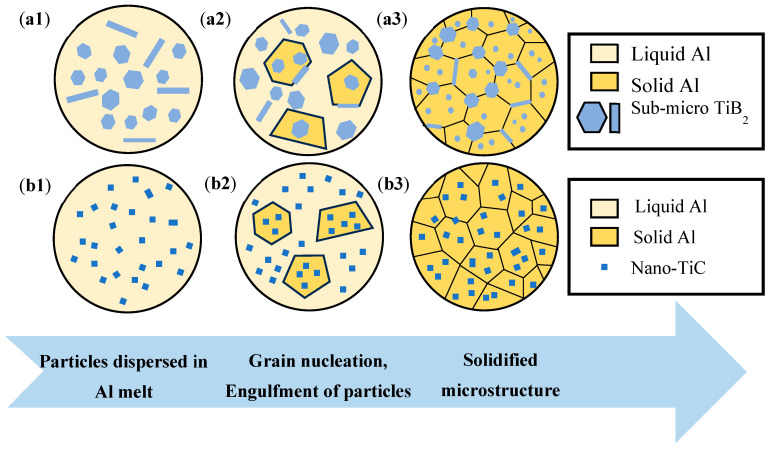
Schematic diagram of microstructure evolution during the solidification of the two alloys: (**a1**–**a3**) TB, (**b1**–**b3**) TC.

In this study, nano-TiC particles of a smaller size were used, and the particles were more evenly dispersed in the grain (as shown in Figure 20b), which could provide more nucleation sites. The diffusion coefficients of particles of different sizes in an Al matrix are different. Stokes–Einstrin diffusion coefficients are given as follows [44]:

(3) $$D=\frac{kT}{6\pi \mu r}$$

where *D* is the diffusion coefficient, *k* is the Boltzmann constant, *T* is the temperature, *μ* is the solvent viscosity, and *r* is the radius of the diffused particle [44]. Since the composition of the base alloy used and the processing parameters for subsequent preparation are all the same, the values of *T* and *μ* for the two alloys in Equation (3) can be approximately equal here. According to Equation (3), it can be concluded that the diffusion coefficient *D* of particles in liquid metal increases with the decrease of particle diameter *r*, indicating that nano-TiC particles are more easily dispersed in the material, with a more uniform distribution and a better thinning effect. Figure 21 shows the microstructure evolution during the solidification of the TB and TC. At the initial stage of solidification, both particles can form crystal nuclei by adsorbing melt atoms (Figure 21(a1,b1)). However, with the gradual solidification, the grains grow gradually, and TiB_2_ particles can optimally drive rapid lateral expansion through the interface to form multi-oriented equiaxed crystals, while TiC realizes restricted growth depending on dynamic curvature regulation (Figure 21(a2,b2)) [45,46]. At the later stage of solidification, TiB_2_ particles are distributed in large quantities on the grain boundary pinning dislocations, hindering grain growth, while TiC particles are dispersed in the grain, providing heterogeneous nucleation sites and promoting nucleation (Figure 21(a3,b3)).

### 4.2. Comparison of the Strengthening Contributions of Two Different Particle-Modified Alloys

The mechanical property results of the TB and TC suggests that the UTS and YS of the AD-TB alloy and AD-TC alloy are similar, but the UTS and YS of the T6-TC alloy are significantly higher than those of T6-TB. Therefore, to understand the strength difference between the two alloys modified by TiB_2_ and TiC particles, it is necessary to explore and compare the strengthening mechanisms and strengthening contributions of the two alloys in as-deposited and T6-treated conditions. Generally, the strengthening mechanisms of the Al alloy mainly include solid solution strengthening ($\sigma_{ss}$), grain boundary strengthening ($\sigma_{GB}$), dislocation strengthening ($\sigma_{dis}$), and precipitation strengthening ($\sigma_{p}$), and the yield strength of the alloy $\left(\sigma_{s}\right)$ can be summarized as follows [47,48,49]:

(4) $$\sigma_{s}=\sigma_{ss}+\sigma_{GB}+\sigma_{dis}+\sigma_{p}$$

where the intrinsic strength ${(\sigma }_{0})$ is included in the $\sigma_{GB}$.

i. Solid solution strengthening ($\sigma_{ss}$)

The solid solution strengthening originates from lattice distortion of the matrix due to the solution of the alloying elements (such as Mg and Cu elements, etc.), which can be calculated by the following formula [50]:

(5) $$\sigma_{ss}=\sum_{i}k_{i}C_{i},(i=Mg,Cu)$$

where $C_{i}$ is the concentration of the *i*-th solute and $k_{i}$ is the proportionality factor of the *i*-th solute: *k_Mg_* ≈ 18.6 MPa (wt.%) [51], *k_Cu_* ≈ 13.8 MPa (wt.%) [52]. The calculated contribution values of solid solution strengthening to the alloys are as follows: the AD-TB is 10.1 MPa, the AD-TC is 8.5 MPa, the T6-TB is 5.5 MPa, and the T6-TC alloy is 5.3 MPa.

ii. Grain boundary strengthening ($\sigma_{GB}$)

Grain boundary strengthening ($\sigma_{GB}$) plays an important role in the strengthening mechanism of the Al alloy. The grain sizes of the as-deposited and heat-treated TB and TC alloys are similar and are all within the effective range of the classic Hall–Petch formula. The strengthening contribution of the grain boundary strengthening can be calculated by the following formula [52]:

(6) $$\sigma_{GB}=\sigma_{0}+k_{1}D_{GB}^{-0.5}$$

where *σ₀* is the intrinsic strength of pure aluminum, which is usually 20 MPa [51]; *k*_1_ is a constant (for Al, *k*₁ = 0.15 MPa·m^−0.5^) [53]; and *D* is the grain size. The calculated contribution of grain boundary strengthening to the alloys are obtained as follows: the AD-TB is 64.8 MPa, the AD-TC is 67.7 MPa, the T6-TB is 65.0 MPa, and the T6-TC is 61.4 MPa.

iii. Dislocation strengthening ($\sigma_{dis}$)

Dislocation strengthening ($\sigma_{dis}$) is an essential component of the alloy strengthening mechanism. During the WAAM of Al-Mg-Si alloys, numerous dislocations are generated due to thermal cycling and residual stress. The strengthening contribution of the dislocation strengthening can be calculated as follows [52]:

(7) $$\sigma_{dis}=M\alpha \mu_{Al}b\rho^{\frac{1}{2}}$$

where Taylor factor *M* is 3.06, the FCC material constant *α* is 0.2, the shear modulus *μ_Al_* of the aluminum base material is 26.2 GPa, *b* represents the Burgers vector (typically 0.286 nm for aluminum alloys), and *ρ* is the line defect density. The calculated contribution values of the dislocation strengthening to the alloy are obtained as follows: the AD-TB is 5.9 MPa, the AD-TC is 4.8 MPa, the T6-TB is 2.4 MPa, and the T6-TC is 2.3 MPa. These results imply that the effect of the dislocation strengthening on the strengthening contribution is very limited in the present work.

iv. Precipitation strengthening ($\sigma_{p}$)

The precipitation strengthening plays an important role in the strengthening contribution of the T6-treated alloys. Generally, the strengthening contribution of the precipitation strengthening can be calculated by the following formula [47]:

(8) $$\sigma_{p}=M\frac{0.4Gb}{\pi (\sqrt{\frac{\pi }{f_{v}}}-2)\bar{R}}\cdot \frac{ln(2\tilde{R}/b)}{\sqrt{1-v}}$$

where *M* is the texture factor of aluminum, with a value of 3.06; *G* is the shear modulus of the α-Al base, with a value of 26.2 GPa; *v* is Poisson’s ratio, with a value of 0.34; $\tilde{R\;}=\sqrt{\left(2/3\right)}\cdot R$, where *R* is the average radius of the precipitate grains; *f_v_* is the volume fraction of the precipitate grains; and *b* is the *B* vector, and for face-centered cubic alloys, *b* = 0.286 nm. The calculated contribution values of the precipitation strengthening to the alloys are obtained as follows: the T6-TB is 182.0 MPa and the T6-TC is 232.2 MPa.

**Figure 22:**
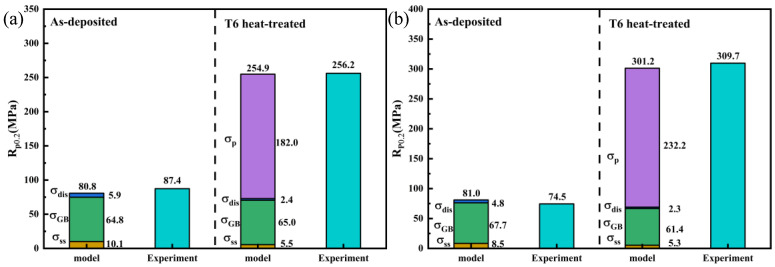
Experimental and model calculation values for yield strength (R_p0.2_) of the as-deposited and T6-treated alloy samples: (**a**) TB, (**b**) TC.

In summary, the strengthening contribution of the grain boundary strengthening, solid solution strengthening, precipitation strengthening and dislocation strengthening to the two alloys in as-deposited and T6-treated conditions is summarized as shown in Figure 22. The detailed calculated $\sigma_{s}$ values are as follows: the AD-TB is 80.8 MPa, the AD-TC is 81.0 MPa, the T6-TB is 254.9 MPa, and the T6-TC is 301.2 MPa. These values are very close to the corresponding experimental values shown in Figure 14 and Figure 15. The results of Figure 22 suggest that the strength difference between two T6-treated alloys is mainly caused by precipitation strengthening, which is associated with the characteristics (i.e., size, number) of the precipitates. In fact, there are significant differences in the precipitation characteristics between TB and TC (Figure 12 and Figure 13) under the same solution and aging treatment. It is reasonable to suspect that the effects of these two particle modifications on the precipitation of precipitates are different, but this is beyond the scope of this work and can be further investigated in the future.

## 5. Conclusions

In this work, TiB_2_ and TiC particles were separately introduced to modify an Al-Mg-Si alloy to enable high-strength WAAM with retained ductility, overcoming the traditional hot cracking issue and strength–ductility tradeoff. This work also offers a scalable pathway for large-scale, crack-free WAAM of lightweight structures for used in the automotive, aerospace and other fields. The main conclusions are summarized as follows:

(1) The modification of Al-Mg-Si alloys with both TiB_2_ and TiC particles completely suppressed the hot cracks that are found in commercial Al-Mg-Si alloys fabricated by WAAM due to the transformation from columnar grains to fine equiaxed grains with an average size of around 10 μm.
(2) The UTS and YS of the AD-TB and AD-TC are similar, but the EL of the latter is higher due to its low porosity. Meanwhile, the UTS (353.7 ± 5.0 MPa) and YS (309.7 ± 1.9 MPa) of the T6-TC are significantly higher than those (300.8 ± 2.7 MPa and 256.2 ± 2.8 MPa, respectively) of the T6-TB.
(3) The fatigue life of the T6-TB and the T6-TC are 4.14 × 10^4^ cycles and 9.05 × 10^4^ cycles, respectively. The fatigue resistance of the T6-TC is better than that of the T6-TB due to its lower porosity and more uniform distribution of TiC particles.

## Figures and Tables

**Table 1:** The nominal chemical composition of the filler wire (wt.%).

Alloys	Mg	Si	Cu	Fe	Cr	Ti	B	C	Al
Al-Mg-Si	1.1	0.6	0.25	0.14	0.15	/	/	/	Bal.
TB	1.1	0.6	0.25	0.14	0.15	0.87	0.39	/	Bal.
TC	1.1	0.6	0.25	0.14	0.15	0.87	/	0.22	Bal.

**Table 2:** The printing parameters for the WAAM process.

Designation	WAAM Deposition Parameters
Wire feed speed (m/min)	6.5
Current (A)	144
Voltage (V)	18
Shileding gas flow rate (L/min)	25
Filling speed (mm/s)	10

**Table 3:** Comparison of mechanical properties between this work and previous works on WAAM-fabricated Al-Mg-Si alloys.

Alloys	Ultimate Tensile Strength *R_m_* /MPa(Stedv)	Yield Strength *R_P_*_0.2_/MPa (Stedv)	Elongation *A*_5_/(%) (Stedv)
Al-0.48Mg-0.54Si-0.25Ti-0.04B [21]	283.5 ± 2.6	262.8 ± 1.7	5.9 ± 0.8
Al-0.87Mg-1.05Si [22]	344 ± 43	189 ± 38	/
Al-0.96Mg-0.58Si-0.23Cu-0.02Fe [24]	336	314	13
T6-TB (this work)	300.8 ± 2.7	256.2 ± 2.8	8.2 ± 0.4
T6-TC (this work)	353.7 ± 2.7	309.7 ± 1.9	5.3 ± 0.4

**Table 4:** Fitted parameters by a Paris model.

Samples	*C*	*m*	*R*	Δ*K_th_*
T6-TB	7.943 × 10^−10^	4.75746	0.926	2.76
T6-TC	1.245 × 10^−11^	6.20116	0.934	4.262

## Data Availability

The original contributions presented in this study are included in the article. Further inquiries can be directed to the corresponding author.
